# SARS-CoV-2 RNA shedding in recovered COVID-19 cases and the presence of antibodies against SARS-CoV-2 in recovered COVID-19 cases and close contacts, Thailand, April-June 2020

**DOI:** 10.1371/journal.pone.0236905

**Published:** 2020-10-29

**Authors:** Chintana Chirathaworn, Manit Sripramote, Piti Chalongviriyalert, Supunnee Jirajariyavej, Phatharaporn Kiatpanabhikul, Jatuporn Saiyarin, Chuleekorn Soudon, Orawan Thienfaidee, Thitisan Palakawong Na Ayuthaya, Chantapat Brukesawan, Dootchai Chaiwanichsiri, Duangnapa Intharasongkroh, Nasamon Wanlapakorn, Jira Chansaenroj, Jiratchaya Puenpa, Ritthideach Yorsaeng, Arunee Thitithanyanont, Rungrueng Kitphati, Anek Mungaomklang, Pijaya Nagavajara, Yong Poovorawan

**Affiliations:** 1 Center of Excellence in Clinical Virology, Department of Pediatrics, Faculty of Medicine, Chulalongkorn University, Bangkok, Thailand; 2 Medical Service Department, Bangkok Metropolitan Administration, Bangkok, Thailand; 3 Taksin Hospital, Medical Service Department, Bangkok Metropolitan Administration, Bangkok, Thailand; 4 Charoenkrung Pracharak Hospital, Medical Service Department, Bangkok Metropolitan Administration, Bangkok, Thailand; 5 Klang General Hospital, Medical Service Department, Bangkok Metropolitan Administration, Bangkok, Thailand; 6 Sirindhorn Hospital, Medical Service Department, Bangkok Metropolitan Administration, Bangkok, Thailand; 7 Ratchaphiphat Hospital, Medical Service Department, Bangkok Metropolitan Administration, Bangkok, Thailand; 8 Public Health Center 28, Health Department, Bangkok Metropolitan Administration, Bangkok, Thailand; 9 Public Health Center 26, Health Department, Bangkok Metropolitan Administration, Bangkok, Thailand; 10 National Blood Center, Thai Red Cross Society, Bangkok, Thailand; 11 Department of Microbiology, Faculty of Science, Mahidol University, Nakhon Pathom, Thailand; 12 Institute for Urban Disease Control and Prevention, Department of Disease Control, Ministry of Public Health, Bangkok, Thailand; 13 Office of the Permanent Secretary for the Bangkok Metropolitan Administration, Bangkok, Thailand; Ben-Gurion University of the Negev, UNITED STATES

## Abstract

Coronavirus disease 2019 (COVID-19) is caused by the severe acute respiratory syndrome coronavirus 2 (SARS-CoV-2). Although Thailand has been fairly effective at controlling the spread of COVID-19, continued disease surveillance and information on antibody response in recovered patients and their close contacts remain necessary in the absence of approved vaccines and antivirals. Here, we examined 217 recovered COVID-19 patients to assess their viral RNA shedding and residual antibodies against SARS-CoV-2. We also evaluated antibodies in blood samples from 308 close contacts of recovered COVID-19 patients. We found that viral RNA remained detectable in 6.6% of recovered COVID-19 cases and up to 105 days. IgM, IgG, and IgA antibodies against SARS-CoV-2 were detected in 13.8%, 88.5%, and 83.4% of the recovered cases 4–12 weeks after disease onset, respectively. Higher levels of antibodies detected were associated with severe illness patients experienced while hospitalized. Fifteen of the 308 contacts (4.9%) of COVID-19 cases tested positive for IgG antibodies, suggesting probable exposure. Viral clearance and the pattern of antibody responses in infected individuals are both crucial for effectively combating SARS-CoV-2. Our study provides additional information on the natural history of this newly emerging disease related to both natural host defenses and antibody duration.

## Introduction

Coronavirus disease 2019 (COVID-19) is caused by the severe acute respiratory syndrome coronavirus 2 (SARS-CoV-2), which first emerged in 2019 and has since spread globally. SARS-CoV-2 is an enveloped, positive-sense single-stranded RNA virus of the family *Coronaviridae* and genus *Betacoronavirus*. It is genetically related to the previously identified severe acute respiratory syndrome coronavirus (SARS-CoV) and the Middle East severe respiratory syndrome coronavirus (MERS-CoV) [1–3].

On March 11, 2020, the World Health Organization (WHO) declared the coronavirus outbreak to be a pandemic. As of October 5, 2020, over 35 million cases of COVID-19 had been reported worldwide, resulting in more than 1 million deaths [4]. While in Thailand alone, approximately 3,590 confirmed COVID-19 cases with 59 deaths had been reported [5]. The first case of COVID-19 was detected in the capital city Bangkok in mid-January 2020. Although early infection was associated with international travelers, rapid increases in reported COVID-19 cases were domestic infections associated with superspreading events at pubs and bars and at a Thai boxing stadium.

SARS-CoV-2-infected individuals often show no or mild symptoms, but severe infection can result in pneumonia and respiratory failure [6]. Real-time reverse-transcription polymerase chain reaction (RT-PCR) is commonly used to detect viral RNA in the laboratory in order to diagnose COVID-19. Viral RNA is detectable in recovered patients, although the duration of viral RNA shedding varied among infected patients and can last at least 6 weeks [7–11]. Some studies have suggested that patients with severe symptoms possessed higher viral loads and longer viremia (>10 days) than patients with mild symptoms (<10 days) [12].

The detection of IgM and IgG antibodies against various SARS-CoV-2 proteins is increasingly utilized [13–15]. The presence of IgM antibodies can be concurrent with acute infection, while detectable IgG antibodies can suggest convalescence or immunity. In addition, secretory IgA antibodies comprises the mucosal immunity necessary to combat infection, although excess IgA immune complexes can lead to uncontrolled cell activation resulting in tissue damage [16, 17]. Due to the limited knowledge on the persistence of different antibody levels following SARS-CoV-2 infection, we aimed to evaluate the immunoglobulin levels and the duration of viral shedding in recovered Thai COVID-19 patients and their close contacts. Investigating antibody status in infected and suspected COVID-19 cases may provide additional insight into the role of immunity in disease recovery and transmission.

## Materials and methods

### Clinical samples

The study protocol was approved by the Institutional Ethics Committee of the Bangkok Metropolitan Administration (BMA) (IRB No. M001h/63_Exp). The IRB waived the need for written consent because samples were obtained through routine preventive measures as part of the public health response to COVID-19 and were de-identified during laboratory analysis. These convenient samples were obtained from recovered laboratory-confirmed COVID-19 patients (n = 207) and individuals who came into close contacts with the patients (n = 308). Patients were previously admitted to either hospitals or public health centers under the BMA and were followed for clinical care thereafter. Although domestic SARS-CoV-2 infection began in early March, 2020 and ended in late April 2020, this study involved recovered patients and close contacts identified between April-June 2020 who consented verbally to be interviewed and to provide nasopharyngeal (NP) swabs and blood samples for analysis. Patient medical records were reviewed by clinicians and health examination was provided at the time of clinical follow-up. Asymptomatic COVID-19 cases were identified in part by the date of diagnosis. Time (in days) between the onset of symptoms and the day of sample collection varied as sample collection times were not fixed and occurred whenever recovered patients were available to come in for evaluation.

Close contacts were defined as individuals with a history of contact with COVID-19 patients who were previously visited by BMA-affiliated doctors and may either be relatives of COVID-19 patients living in the same household or interacted with patients for a significant amount of time (including healthcare providers to the patients, passengers on the same bus, close friends, co-workers, and neighbors). Close contacts were interviewed and provided health examination in accordance with the BMA quarantine protocol. For close contacts who agreed to provide blood samples for this study, the date of the last contact with the case and the date of blood collection were recorded, which varied depending on when they were willing and able to come in. In all, these convenient samples were collected from each recovered cases and close contacts only once. Serial NP swabs were performed only in the cases with prolonged viral shedding.

### Real-time RT-PCR to detect viral RNA

Real-time RT-PCR targeting the RNA-dependent RNA polymerase (RdRp) and envelope (E) gene of SARS-CoV-2 was performed using the LightMix Modular SARS and Wuhan CoV E-gene Kit (TIB MOBIOL, Synheselabor GmbH, Berlin, Germany) in a LightCycler 480 II system according to the manufacturer’s instructions (Roche Diagnostics International Ltd., Rotkreuz, Switzerland). Results were reported as detectable (Ct values ≤38) or undetectable (Ct values ≥40). Samples yielding Ct values in the intermediate range were repeated and were considered positive if the second Ct values remained ≤40.

### Detection of antibodies against SARS-CoV-2 by enzyme-linked immunosorbent assay (ELISA)

Recovered patients were evaluated for IgM, IgG, and IgA antibodies against SARS-CoV-2, while close contacts were evaluated for IgG alone. IgM antibody levels were measured using the MAGLUMI 2000 fully automated chemiluminescent analytical system (Snibe, Shenzhen, China) according to the manufacturer’s instructions. Values were reported as arbitrary unit per milliliter (AU/mL). Determination of IgG and IgA antibodies was performed using an automated ELISA system (EUROIMMUN, Lübeck, Germany) according to the manufacturer’s instructions. The optical density (OD) at 450 nm was measured. The resulting sample to cut-off ratio of ≥1.1 was considered seropositive.

### Statistical analysis

The association between antibody levels and clinical characteristics was analyzed using the Mann-Whitney *U*-test and chi-square test. The association between age group and clinical characteristics was examined by the chi-square test. The difference between the numbers of contacts positive for IgG antibodies against SARS-CoV-2 in each group of close contacts was determined using the chi-square test. A *p*-value <0.05 was considered statistically significant.

## Results

Among recovered patients, there were more women than men (57.6% vs. 42.4%) and women were slightly younger in age (median = 31 years) (Table 1). Median age of close contacts were similar to that of recovered cases. There were roughly equal number of men and women among the close contacts. Review of the 217 hospital records of patients who recovered from COVID-19 showed that patients experienced no symptoms (4/217), mild symptoms (151/217), pneumonia (59/217), and pneumonia requiring tracheal intubation (3/217). Among the 308 close contacts, 118 resided in the same household. Other close contacts were close friends, co-workers, healthcare personnel who took care of the COVID-19 cases, taxi drivers, neighbors, or individuals who lived or performed activities in the same community as the COVID-19 cases.

**Table 1:** Recovered COVID-19 cases and close contacts included in this study and the results of antibody detection.

	Recovered cases	Close contacts
**Total number**	217	308
**Median age in years (IQR)**	33 (25–47)	35 (26–48)
**Gender**		
Number of males (%)	92 (42.4)	159 (51.6)
and median age in years (IQR)	36 (29–48)	34 (25–47)
Number of females (%)	125 (57.6)	149 (48.4)
and median age in years (IQR)	31 (25–45)	37 (27.5–51)
**Clinical History**		
Asymptomatic (%)	4 (1.8)	NA
Mild symptoms (%)	151 (69.6)	
Pneumonia (%)	59 (27.2)	
Pneumonia requiring intubation (%)	3 (1.4)	
**Time from patient symptom onset to sample collection (**days)	range 28–142	NA
	median 54 (IQR 45–61)	
**Time from the last contact with patient to sample collection (**days)	NA	range 1–128
		median 61.5
		(IQR 47.5–67)
**IgM antibody detection**		
Number of positive cases (%)	30/217 (13.8)	ND
Time from patient symptom onset to	range 34–67	
sample collection for positive cases (days)	median 51 (IQR 39–57.3)	
**IgG antibody detection**		
Number of positive cases (%)	192/217 (88.5)	15/308 (4.9)
Time from symptom onset to	range 28–142	
sample collection for positive case (days)	median 53 (IQR 44–60)	
**IgA antibody detection**		
Number of positive cases (%)	181/217 (83.4)	ND
Time from symptom onset to	range 28–142	
sample collection for positive cases (days)	median 53 (IQR 44–60)	

NA, not applicable; ND, not done.

### Detection of SARS-CoV-2 RNA in recovered COVID-19 cases

Only 212 NP swabs from 217 recovered COVID-19 cases were available for analysis. From these, 14 (6.6%) yielded detectable viral RNA by real-time RT-PCR (age range 16–67). Two samples showed Ct values <30 (28.4 and 29.6). The Ct values of the remaining 12 cases ranged between 30.2 and 37.7. The time between the day of symptom onset and sample collection ranged from 36–105 days (median = 54 days). We were able to obtain and test serial NP swab samples from 12 of the 14 recovered cases (2 of whom could not be followed up) in order to evaluate how long the viral RNA remains detectable by real-time RT-PCR. Viral RNA was detected for up to 15 weeks after the onset of COVID-19 symptoms.

### Antibodies against SARS-CoV-2 in recovered COVID-19 cases

**Fig 1:**
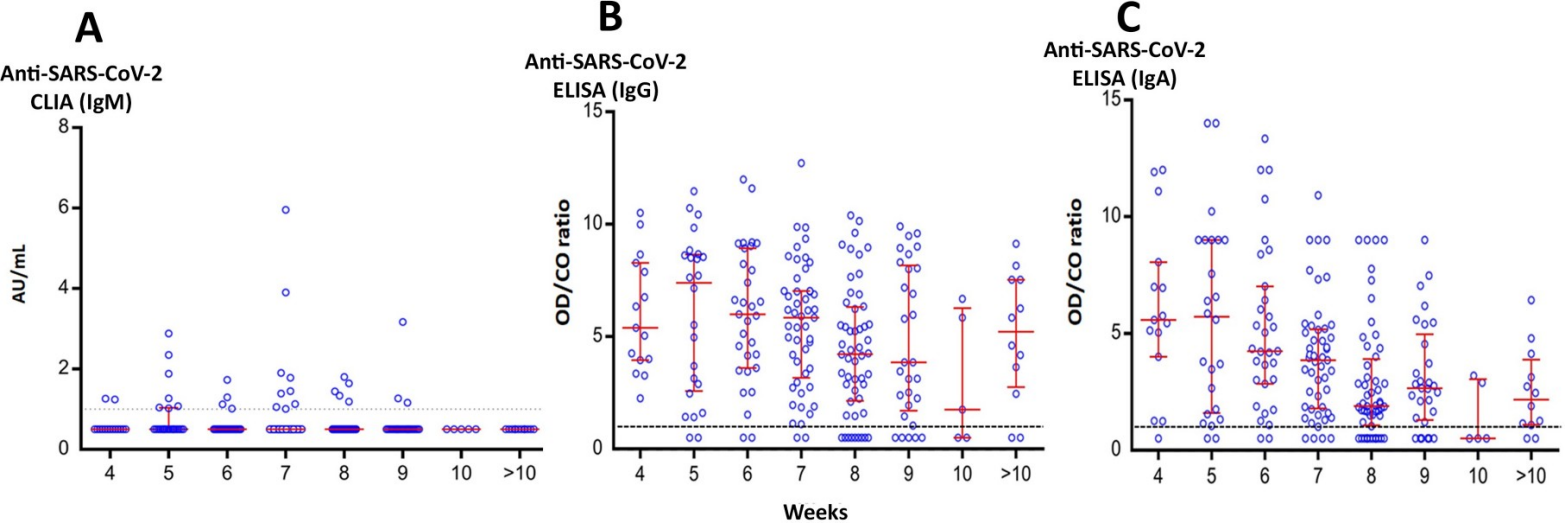
Antibodies against SARS-CoV-2 in recovered COVID-19 cases. IgM (A), IgG (B), and IgA (C) antibody levels were stratified according to the number of weeks between the day of symptom onset and the day of sample collection. Horizontal lines represent the upper and lower interquartile range (IQR) and the median value.

The levels of IgM, IgG, and IgA antibodies against SARS-CoV-2 in blood samples from 217 recovered cases were determined according to the time in weeks between the day of symptom onset and the day blood sample was collected. Thirty samples tested positive for IgM (13.8%), all of which also tested positive for IgG and IgA (Fig 1A). These IgM antibodies remained detectable for up to 2 months in some cases (Table 1). There were 192 (88.5%) IgG-positive and 181 (83.4%) IgA-positive samples, of which 150 (69.1%) tested positive for both IgG and IgA (Fig 1B and 1C). Both IgG and IgA antibodies were detectable for up to 20 weeks (S1 Table). The time between the day of the first symptom onset and blood sample collection varied from 28–142 days.

**Fig 2:**
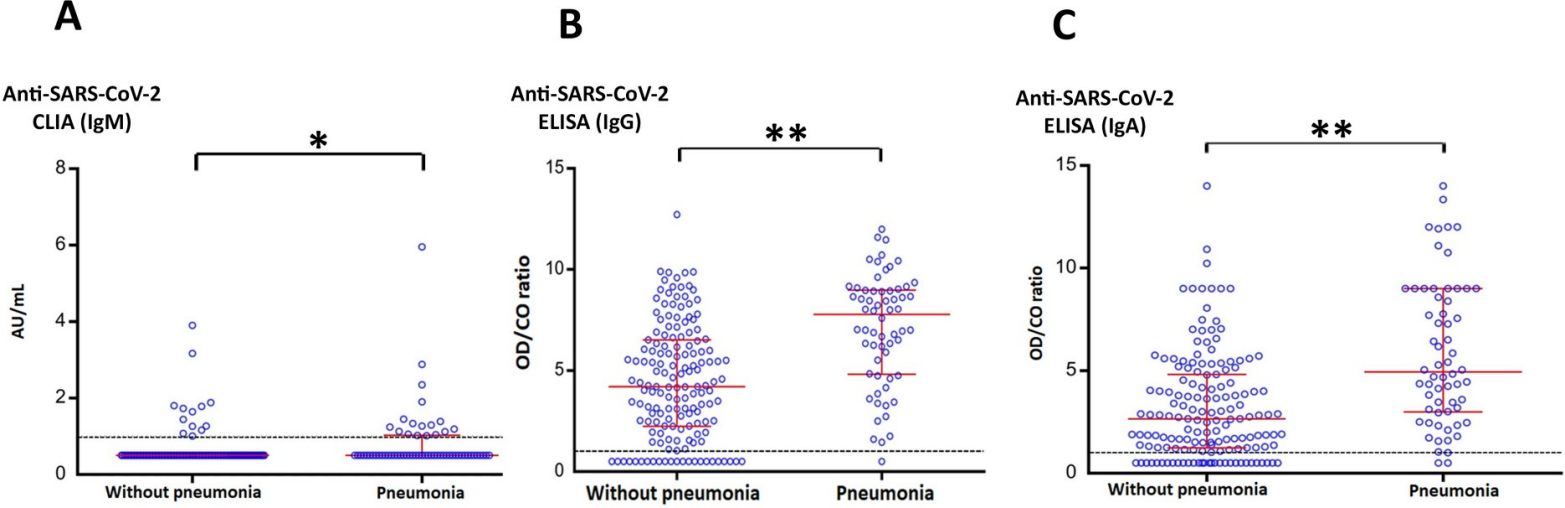
The association between the antibodies against SARS-CoV-2 and symptoms of recovered COVID-19 cases. The levels of IgM (A), IgG (B), and IgA (C) antibodies against SARS-CoV-2 in recovered COVID-19 cases with and without pneumonia are represented as dots. Bars represent median values (middle line) and 1× the upper and lower interquartile range (IQR) (upper and lower lines). The levels of IgM, IgG, and IgA antibodies against SARS-CoV-2 were significantly higher in patients with pneumonia than in those without pneumonia. * *p* = 0.0002, ** *p* < 0.00001.

Since COVID-19 symptoms varied, we next examined possible association between disease severity in patients and antibody levels. Recovered patients were grouped into those with pneumonia (62/217 cases, median age = 36.5) and without pneumonia (asymptomatic and mild symptoms, 155/217 cases, median age = 32) (Fig 2). The ages of cases were significantly different between these two groups (chi-square *p* = 0.027). Pneumonia was associated with increasing age (*p* = 0.001) (S2 Table). There were more patients with history of pneumonia who demonstrated immunoglobulin positivity than patients without pneumonia (chi-square *p* ≤ 0.1) (S3 Table). Their antibody levels and the IQR were also generally higher (*p* ≤ 0.001). When antibody levels detected in recovered COVID-19 patients were stratified by time after onset of symptoms (<6 weeks, 6–8 weeks, and >8 weeks), IgM levels gradually declined, while IgG and IgA levels remained significantly higher among patients with pneumonia (S4, S5, and S6 Tables).

### Antibody detection in close contacts of SARS-CoV-2 patients

**Fig 3:**
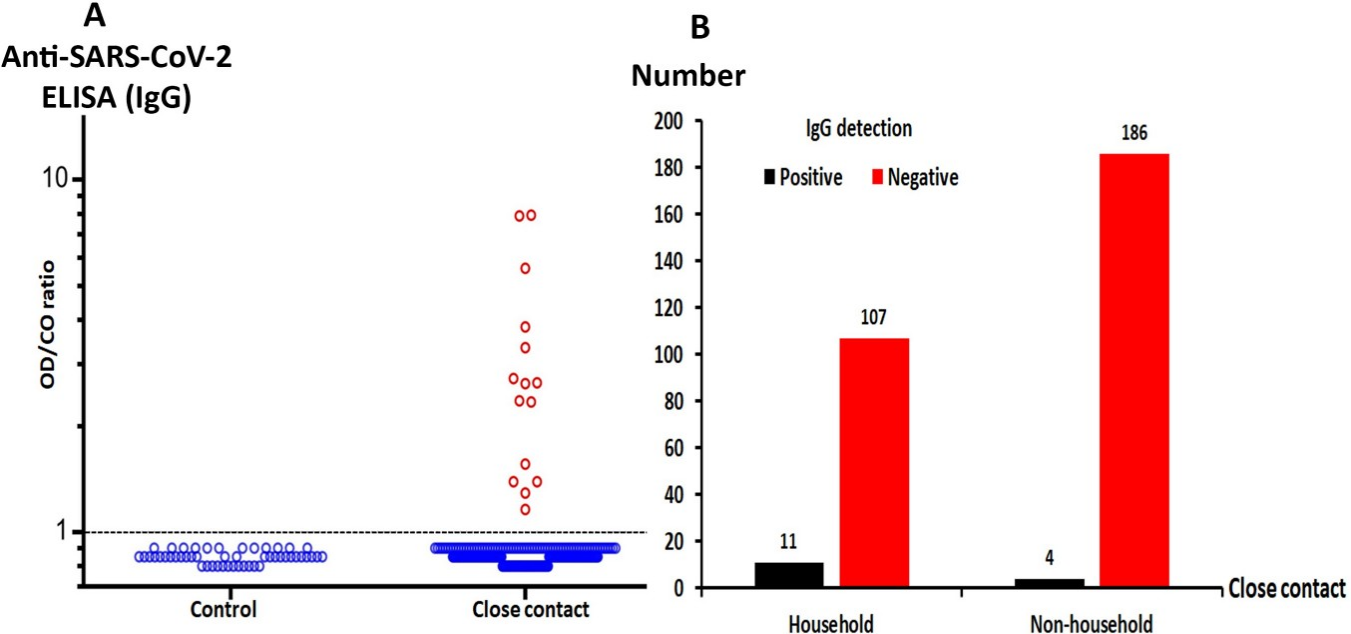
IgG antibody against SARS-CoV-2 among close contacts of COVID-19 cases. (A) IgG antibodies against SARS-CoV-2 in blood samples from 308 close contacts of COVID-19 cases compared to 50 healthy controls collected in 2018 (prior to the emergence of COVID-19). (B) IgG detection among household and non-household close contacts.

Among 308 close contacts of COVID-19 patients who were subjected to IgG evaluation, 15 (4.9%) demonstrated detectable IgG levels (median = 2.6, IQR 1.4–3.8) (Fig 3A). Eleven of these close contacts presented mild symptoms of upper respiratory tract infection. These contacts were placed under investigation and quarantined for 14 days according to the protocol. In this study, 118 of the 308 close contacts lived in the same household as the patient, 11 of whom tested positive for IgG (9.3%) (Fig 3B). Four others who demonstrated detectable IgG were non-household members of the COVID-19 cases (4/190, 2.1%) (chi-square, *p* = 0.015). This result suggests that household contacts represent a high-risk group for disease exposure.

## Discussion

The number of cases of COVID-19 has increased rapidly worldwide since the emergence of SARS-CoV-2 infection in December 2019. Development of laboratory diagnostics and potential treatments had been urgently evaluated, while unexpected clinical manifestation associated with the duration of infection continues to emerge. Several studies have reported the kinetics of antibody production in COVID-19 patients while undergoing medical care. Our study examined viral RNA shedding, antibody responses in COVID-19 cases and the evidence of viral exposure to close contacts. We chose to follow the recovered cases to investigate the persistence of viral RNA shedding antibodies against SARS-CoV-2. We showed that viral shedding could still be detected in 14/212 (6.6%) of the recovered COVID-19 cases, the longest of which can last for up to 105 days. Since virus-specific IgM typically declines following acute infection, we only observed detectable IgM in 13.8% of recovered cases. In contrast, IgA and IgG antibodies remained detectable in more than 80% of recovered cases. Antibody levels were analyzed according to the time samples were collected after symptom onset and our data showed that the antibody levels gradually declined. However, the kinetics of antibody production and regulation could not be clearly demonstrated due to the limited number of samples in some interval times. Using pneumonia as a surrogate of illness severity, we were able to show that some antibody levels remained high despite the patients having recovered from the disease.

The report of a COVID-19 case in Taiwan showed that viral RNA could be detected on day 5 after disease onset, while IgM antibodies were present between days 11 and 27 [18]. Earlier study reported that IgM and IgG antibodies against SARS-CoV-2 could be detected using ELISA as early as the fourth day after symptom onset with sensitivities of 77.3% for IgM and 83.3% for IgG [19]. Our finding of higher levels of IgG and IgA antibodies, particularly in patients with severe illness, was consistent with a previous study in which samples collected between 6 and 27 days after diagnosis showed earlier and higher seroconversion [20]. Similar findings were previously demonstrated in MERS-CoV infection [21, 22]. A study on SARS-associated coronavirus showed that IgG antibodies persist longer than IgA antibodies and are therefore more suitable for disease surveillance [23]. Furthermore, neutralizing IgM and IgG antibodies could be detected within 9 days of symptom onset and remained detectable on day 20 after symptom onset [24].

Early reports of COVID-19 to describe disease progression and serological response to viral infection have primarily been from various studies conducted in China. Our observation that COVID-19 patients in Thailand who experienced pneumonia demonstrated higher levels of detectable residual immunoglobulins was consistent with a previous Chinese study showing that the concentrations of IgG and IgM appeared to be higher in severe cases of COVID-19 [25]. It was reported that disease severity correlated with longer detectable viral RNA for up to a month after disease onset [26, 27]. We were surprised that we were able to detect viral RNA as long as 105 days after symptom onset in our patient cohort, which suggests possible delayed viral clearance in some recovered patients as a result of the ability for the virus to replicate better and/or immunocompetence status in some people. Although detectable viral RNA among asymptomatic cases do not always correlated with the presence of detectable antibody response, our study found that high antibody titers were associated with the worst clinical manifestations, and detection of viral RNA showed the greatest sensitivity during the first week of symptom onset.

In our study, 4.9% of close contacts had probable viral exposure as evaluated by the presence of virus-specific IgG. This small percentage may have been due to several factors, including reduced exposure of household members to patients once they are hospitalized, avoidance of symptomatic members, and willingness to mitigate transmission risk by frequent handwashing and wearing masks. Not surprisingly, close contacts of COVID-19 patients have been shown to be at risk of infection [28]. However, it is not unusual that close contacts are asymptomatic despite exposure [29].

Evaluation of viral antibodies after COVID-19 patients have recovered provides valuable insight into the potential sustainability of antibody levels sufficient to protect against re-infection. Our data showed that 10 weeks post-onset of symptoms, over 80% of samples in our recovered cohort remained positive for IgG and IgA antibodies. Six samples with the duration after onset over 100 days (104–142 days) were still positive for IgG and IgA antibodies. In about 10% of the recovered cases, IgG and IgA were no longer detectable as early as week 5 after symptom onset, while only around 30% of recovered patients retained IgM antibodies. It would be interesting to further investigate whether the disappearance of antibodies so soon after infection was due to rapid decline or low antibody production. Finally, our study confirmed the association of increased antibody levels with increased severity of patient illness.

This study was limited in part by the number of recovered patients available and their willingness to participate. We did not evaluate antibody levels while patients were still clinically symptomatic. Serial samples collected at defined time points would have been ideal in examining the kinetics of antibody production. Patients in our cohort did not include the very young and the very old, which could have affected the risk of developing severe illness. Our data may not be representative of the overall characteristics of recovered COVID-19 patients elsewhere around the world, but they do provide valuable insight to guide and refine policies to further limit the spread of SARS-CoV-2 infection in this country. If convalescent plasma were to be used for COVID-19 treatment, these data will provide estimates of the required number of recovered patients to be screened and be used to establish the antibody baseline by the National Blood Center towards plasma donation in Thailand.

## Supporting information

S1 Table Antibodies against SARS-CoV-2 in recovered COVID-19 cases according to how long after the onset of COVID-19 symptoms the blood sample was collected. (DOCX)

S2 Table Number of recovered COVID-19 cases with and without pneumonia stratified by age groups. (DOCX)

S3 Table Antibodies against SARS-CoV-2 in recovered COVID-19 cases with and without pneumonia. (DOCX)

S4 Table IgG antibodies in recovered COVID-19 cases with and without pneumonia stratified by how long after onset of COVID-19 symptoms the blood sample was collected. (DOCX)

S5 Table IgA antibodies in recovered COVID-19 cases with and without pneumonia stratified by how long after onset of COVID-19 symptoms the blood sample was collected. (DOCX)

S6 Table IgM antibodies in recovered COVID-19 cases with and without pneumonia stratified by how long after onset of COVID-19 symptoms the blood sample was collected. (DOCX)
