# Supplementary material for: SARS-CoV-2 RNA shedding in recovered COVID-19 cases and the presence of antibodies against SARS-CoV-2 in recovered COVID-19 cases and close contacts, Thailand, April-June 2020
Source: PLoS One. 2020 Oct 29;15(10):e0236905. doi: 10.1371/journal.pone.0236905 (PMC7595404; doi:10.1371/journal.pone.0236905)
Supplement: S1 Table — (DOCX) [file pone.0236905.s001.docx]

**S1 Table. Antibodies against SARS-CoV-2 in recovered COVID-19 cases according to how long after the onset of COVID-19 symptoms the blood sample was collected.**

| Weeks after onset (n) | IgG level | | IgA level | | IgM level | |
| --- | --- | --- | --- | --- | --- | --- |
|  | Positive cases n (%) | Median (IQR) | Positive cases  n (%) | Median (IQR) | Positive cases  n (%) | Median (IQR) |
| week 4 (15)  week 5 (24)  week 6 (31)  week 7 (49)  week 8 (52)  week 9 (29)  week 10 (5)  week > 10 (12) | 15 (100.0)  22 (91.7)  29 (93.6)  47 (95.9)  43 (82.7)  23 (79.3)  3 (60.0)  10 (83.3) | 5.4 (4.0-8.3)  7.4 (2.6-8.7)  6.0 (3.6-8.9)  5.8 (3.2-7.0)  4.2 (2.1-6.3)  3.9 (1.7-8.2)  1.8 (0.2-6.3)  5.2 (2.8-7.5) | 14 (93.3)  21 (87.5)  28 (90.3)  44 (89.8)  39 (75.0)  23 (79.3)  2 (40.0)  10 (83.3) | 5.6 (4-8.1)  5.7 (1.6-8.0)  4.2 (2.8-7.0)  3.9 (1.8-5.2)  1.9 (1.1-3.9)  2.8 (1.3-5.4)  0. 8 (0.3-3.0)  2.6 (1.2-3.9) | 2 (13.3)  7 (29.2)  4 (12.9)  9 (18.4)  5 (9.6)  3 (10.3)  0 (0)  0 (0) | 0.7 (0.6-0.8)  0.8 (0.7-1.0)  0.7 (0.6-0.8)  0.7 (0.6-0.8)  0.6 (0.5-0.8)  0.6 (0.5-0.9)  0.6 (0.5-0.7)  0.6 (0.4-0.7) |
