# Supplementary material for: SARS-CoV-2 RNA shedding in recovered COVID-19 cases and the presence of antibodies against SARS-CoV-2 in recovered COVID-19 cases and close contacts, Thailand, April-June 2020
Source: PLoS One. 2020 Oct 29;15(10):e0236905. doi: 10.1371/journal.pone.0236905 (PMC7595404; doi:10.1371/journal.pone.0236905)
Supplement: S2 Table — (DOCX) [file pone.0236905.s002.docx]

**S2 Table. Number of recovered COVID-19 cases with and without pneumonia stratified by age groups.**

| Age (years) | n | Gender  n (%) | | History with pneumonia  n (%) | |
| --- | --- | --- | --- | --- | --- |
|  |  | Male | Female | Yes | No |
| 2-20 | 15 | 6 (40.0) | 9 (60.0) | 4 (26.7) | 11 (73.3) |
| 21-30 | 74 | 21 (28.4) | 53 (71.6) | 10 (13.5) | 64 (86.5) |
| 31-40 | 50 | 28 (56) | 22 (44) | 15 (30.0) | 35 (70.0) |
| 41-50 | 41 | 18 (43.9) | 23 (56.1) | 16 (39.0) | 25 (61.0) |
| 51-60 | 22 | 11 (50.0) | 11 (50.0) | 8 (36.4) | 14 (63.6) |
| 61-76 | 15 | 8 (53.3) | 7 (46. 7) | 9 (60.0) | 6 (40.0) |
