# Supplementary material for: SARS-CoV-2 RNA shedding in recovered COVID-19 cases and the presence of antibodies against SARS-CoV-2 in recovered COVID-19 cases and close contacts, Thailand, April-June 2020
Source: PLoS One. 2020 Oct 29;15(10):e0236905. doi: 10.1371/journal.pone.0236905 (PMC7595404; doi:10.1371/journal.pone.0236905)
Supplement: S3 Table — (DOCX) [file pone.0236905.s003.docx]

**S3 Table. Antibodies against SARS-CoV-2 in recovered COVID-19 cases with and without pneumonia.**

|  | With pneumonia (n=62) | Without pneumonia (n=155) |
| --- | --- | --- |
| IgG positive, n (%)  IgG negative, n (%)  IgG level, median (IQR) | 61 (98.4)  1 (1.6)  7.8 (4.8-9.0) | 131 (84.5)  24 (15.5)  4.2 (2.2-6.5) |
| IgA positive, n (%)  IgA negative, n (%)  IgA level, median (IQR) | 58 (93.6)  4 (6.5)  5.0 (3.0-8.0) | 123 (79.4)  32 (20.7)  2.7 (1.3-4.9) |
| IgM positive, n (%)  IgM negative, n (%)  IgM level, median (IQR) | 17 (27.4)  45 (72.6)  0.7 (0.6-1.0) | 13 (8.4)  142 (91.6)  0.6 (0.5-0.8) |
