# Supplementary material for: SARS-CoV-2 RNA shedding in recovered COVID-19 cases and the presence of antibodies against SARS-CoV-2 in recovered COVID-19 cases and close contacts, Thailand, April-June 2020
Source: PLoS One. 2020 Oct 29;15(10):e0236905. doi: 10.1371/journal.pone.0236905 (PMC7595404; doi:10.1371/journal.pone.0236905)
Supplement: S4 Table — (DOCX) [file pone.0236905.s004.docx]

**S4 Table. IgG antibodies in recovered COVID-19 cases with and without pneumonia stratified by how long after onset of COVID-19 symptoms the blood sample was collected.**

| Weeks after onset |  | IgG level in cases with pneumonia | | IgG level in cases without pneumonia | | |
| --- | --- | --- | --- | --- | --- | --- |
|  | n | Positive cases n (%) | Median (IQR) | n | Positive cases n (%) | Median (IQR) |
| <6 weeks  6-8 weeks  >8 weeks | 12  31  19 | 12 (100)  30 (96.8)  19 (100) | 8.7 (6.3-10.5)*  7.0 (4.8-8.9)*  7.0 (4.2-9.0)* | 27  56  72 | 25 (92.6)  52 (92.9)  54 (75.0) | 5.0 (2.9-7.7)  4.6 (2.6-6.4)  3.9 (1.2-5.9) |

Asterisk denotes significantly higher IgG levels in this group (p value = 0.003, <0.001, <0.001 at <6, 6-8 and > 8 weeks, respectively).
