# Supplementary material for: SARS-CoV-2 RNA shedding in recovered COVID-19 cases and the presence of antibodies against SARS-CoV-2 in recovered COVID-19 cases and close contacts, Thailand, April-June 2020
Source: PLoS One. 2020 Oct 29;15(10):e0236905. doi: 10.1371/journal.pone.0236905 (PMC7595404; doi:10.1371/journal.pone.0236905)
Supplement: S5 Table — (DOCX) [file pone.0236905.s005.docx]

**S5 Table. IgA antibodies in recovered COVID-19 cases with and without pneumonia stratified by how long after onset of COVID-19 symptoms the blood sample was collected.**

| Weeks after onset |  | IgA level in cases with pneumonia | | IgA level in cases without pneumonia | | |
| --- | --- | --- | --- | --- | --- | --- |
|  | n | Positive cases n (%) | Median (IQR) | n | Positive cases n (%) | Median (IQR) |
| <6 weeks  6-8 weeks  >8 weeks | 12  31  19 | 11 (91.7)  29 (93.6)  18 (94.7) | 8.0 (6.1-11.2)*  4.7 (3.0-8.0)*  4.1 (2.5-6.5)* | 27  56  72 | 24 (88.9)  48 (85.7)  51 (70.8) | 5.0 (1.3-6.6)  3.4 (1.5-5.1)  2.0 (0.7-3.2) |

Asterisk denotes significantly higher IgA levels in this group (p value = 0.007, 0.007, 0.001 at <6, 6-8 and > 8 weeks, respectively).
