# Supplementary material for: SARS-CoV-2 RNA shedding in recovered COVID-19 cases and the presence of antibodies against SARS-CoV-2 in recovered COVID-19 cases and close contacts, Thailand, April-June 2020
Source: PLoS One. 2020 Oct 29;15(10):e0236905. doi: 10.1371/journal.pone.0236905 (PMC7595404; doi:10.1371/journal.pone.0236905)
Supplement: S6 Table — (DOCX) [file pone.0236905.s006.docx]

**S6 Table. IgM antibodies in recovered COVID-19 cases with and without pneumonia stratified by how long after onset of COVID-19 symptoms the blood sample was collected.**

| Weeks after onset |  | IgM level in cases with pneumonia | | IgM level in cases without pneumonia | | |
| --- | --- | --- | --- | --- | --- | --- |
|  | n | Positive cases n (%) | Median (IQR) | n | Positive cases n (%) | Median (IQR) |
| <6 weeks  6-8 weeks  >8 weeks | 12  31  19 | 6 (50%)  9 (29.0)  2 (10.5) | 1.0 (0.7-1.2)*  0.7 (0.6-1.1)  0.7 (0.6-0.9) | 27  56  72 | 3 (11.1)  4 (7.1)  6 (8.3) | 0.7 (0.6-0.8)  0.7 (0.6-0.8)  0.6 (0.5-0.7) |

Asterisk denotes significantly higher IgM levels in this group (p value = 0.01).
